# OLDER AGE IS (STILL) ASSOCIATED WITH MENTAL HEALTH BENEFITS IN JUNE–JULY 2021 OF THE COVID-19 PANDEMIC

**DOI:** 10.1093/geroni/igac059.1955

**Published:** 2022-12-20

**Authors:** Amanda Chappell, Kelly Smith, JoNell Strough

**Affiliations:** West Virginia University, Morgantown, West Virginia, United States; WVU, Morgantown, West Virginia, United States; WVU, Morgantown, West Virginia, United States

## Abstract

In the early months of the COVID-19 pandemic, older age was associated with less anxiety and depression (Bruin de Bruin, 2021). Similar results were found for data collected during the June-July 2020 spike in cases (Smith et al., 2021). Theorists have suggested that benefits of age for well-being may be reduced when stressors are prolonged and unavoidable (Charles, 2010). Here, we investigated whether older age continued to be protective in June-July 2021, when vaccines had become widely available, but the pandemic persisted. Secondary data analysis was conducted from the Understanding America Study, based on n=5,535 (M=52.69 yrs., SD=16.04) participants who responded to online self-report surveys. Participants reported symptoms of anxiety and depression (assessed by the Patient Health Questionnaire, PHQ-4), engagement in protective behaviors (e.g., wearing a mask), and coping strategies (e.g., getting extra exercise). Multiple regression analyses predicted anxiety and depression from age, coping strategies, and protective behaviors, controlling for marital status, gender, and income. Coping through exercise and calling family/friends were significantly associated with less anxiety and depression, whereas coping by using social media and engaging in protective behaviors was significantly associated with more anxiety and depression. The harmful effects of protective behaviors may reflect the people engaging in these strategies most often are also those most worried about COVID-19. Even after accounting for coping strategies and protective behaviors, older age was still associated with fewer symptoms of anxiety and depression. Implications of older adults’ resilience in the face of a prolonged stressor for promoting mental health are discussed.

